# The photocurrent response of human cones is fast and monophasic

**DOI:** 10.1186/1471-2202-7-34

**Published:** 2006-04-20

**Authors:** JH van Hateren, TD Lamb

**Affiliations:** 1 Department of Neurobiophysics, University of Groningen, Groningen, The Netherlands; 2 Division of Neuroscience, John Curtin School of Medical Research, and ARC Centre of Excellence in Vision Science, Australian National University, Canberra, Australia

## Abstract

**Background:**

The precise form of the light response of human cone photoreceptors *in vivo *has not been established with certainty. To investigate the response shape we compare the predictions of a recent model of transduction in primate cone photoreceptors with measurements extracted from human cones using the paired-flash electroretinogram method. As a check, we also compare the predictions with previous single-cell measurements of ground squirrel cone responses.

**Results:**

The predictions of the model provide a good description of the measurements, using values of parameters within the range previously determined for primate retina. The dim-flash response peaks in about 20 ms, and flash responses at all intensities are essentially monophasic. Three time constants in the model are extremely short: the two time constants for inactivation (of visual pigment and of transducin/phosphodiesterase) are around 3 and 10 ms, and the time constant for calcium equilibration lies in the same range.

**Conclusion:**

The close correspondence between experiment and theory, using parameters previously derived for recordings from macaque retina, supports the notion that the electroretinogram approach and the modelling approach both provide an accurate estimate of the cone photoresponse in the living human eye. For reasons that remain unclear, the responses of isolated photoreceptors from the macaque retina, recorded previously using the suction pipette method, are considerably slower than found here, and display biphasic kinetics.

## Background

The precise form of the light response of human cone photoreceptors *in vivo *has not been established with certainty. Measurements have been made from single cone cells isolated from the macaque retina using the suction pipette technique, and in these experiments the dim-flash time-to-peak was about 50 ms and the responses exhibited biphasic kinetics [1]. On the other hand, measurements made in the living human eye using the paired-flash ERG technique have found a much faster time-to-peak for the dim-flash response, of about 20 ms [2]. Most recently, van Hateren [3] has analyzed macaque horizontal cell responses recorded in a retina-RPE-choroid preparation [4,5], using a theoretical model combining cone transduction and synaptic transmission, and he has extracted values for transduction parameters that permit the cone photocurrent response to be simulated. Here we show that this model for macaque cone transduction provides a good description of the cone response kinetics estimated from the human ERG, supporting the occurrence of a very rapid peak, and indicating that the flash response has monophasic kinetics.

**Figure 1:**
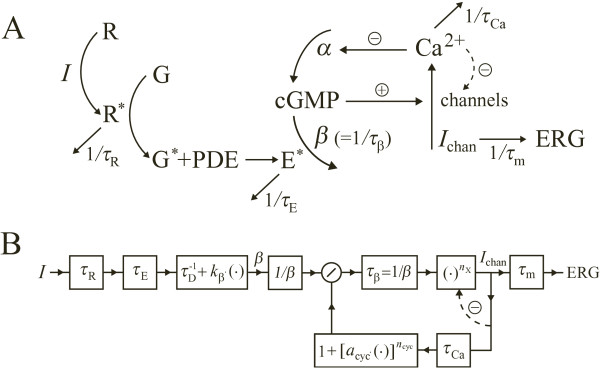
**Reaction scheme and model for the generation of photocurrent in human cones**. A. Photopigment R is converted by light *I* into activated photopigment R*, which is inactivated with rate constant 1/τ_R_. R* activates a G-protein (transducin) which subsequently forms an activated complex with PDE. The activated transducin-PDE complex is inactivated with rate constant 1/τ_E_; it hydrolyses cGMP with rate constant β. CNG channels in the membrane, opened under control of cGMP, admit an ionic current *I*_chan_, part of which is carried by Ca^2+ ^ions. Ca^2+^, which is removed with rate constant 1/τ_Ca_, regulates the production rate α of cGMP via guanylyl cyclase (GC). Ca^2+^ also exerts a direct inhibitory influence on the CNG channels. Part of the ionic current *I*_chan _charges the membrane of the cone outer segment (with membrane time constant τ_m_), and the remainder of *I*_chan _flows into the cone inner segment. In the intact eye the extracellular flow of this circulating current produces an extracellular voltage drop, which is measured as the cone contribution to the ERG (as in Fig. 2). In single-cell experiments the extracellular flow of current may be measured using a suction pipette (as in Fig. 3). B. The boxes with τ_(·) _represent first-order low-pass filters. The rate constant of cGMP hydrolysis consists of a dark rate constant, 1/τ_D_, plus a light-dependent component with scaling determined by the gain factor *k*_β_. The nonlinear differential equation describing cGMP hydrolysis can be understood as a static nonlinearity 1/β followed by a low-pass filter with time constant τ_β _= 1/β [3]. The calcium feedback loop is governed by *n*_X_, the apparent Hill coefficient of cGMP activation of the CNG channels (including the effect of a local calcium feedback, indicated by the dashed line), and by *n*_cyc_, the Hill coefficient of GC deactivation; *a*_cyc _is a scaling constant.

The reaction cascade underlying the cone phototransduction module of [3] conforms with other recent descriptions (see [6]), and is shown in Fig. 1A. This cascade can be translated into the mathematical scheme, or system model, illustrated in Fig. 1B; for further details, see [3] and Methods.

Each photon of light absorbed by a visual pigment molecule triggers formation of the activated transducin-PDE complex (E*), with double integrating kinetics defined by the lifetimes of active pigment (τ_R_) and active transducin-PDE (τ_E_). To minimize the number of parameters in the model, the gain of E* activation can be combined with the catalytic efficacy of E*, to give a gain factor *k*_β _that specifies the increase in rate constant of cGMP hydrolysis per unit of illumination.

The hydrolysis of cGMP leads to closure of cyclic nucleotide-gated (CNG) channels, and reduction of the channel current (*I*_chan_), part of which is carried by calcium ions. The resulting drop in calcium concentration activates guanylyl cyclase (GC) activity, tending to restore the level of cGMP. Whenever *I*_chan _changes, a component of the current flows locally, to charge the capacitance of the outer segment membrane, and this component is not recordable externally. Instead the observable component of current, which flows into the cone inner segment, is low-pass filtered with time constant τ_m_. The return flow of this circulating current causes an extracellular voltage drop that contributes to the ERG. In this formulation we are ignoring the effects of the calcium exchange current, because further analysis indicates that it provides only slight additional filtering.

## Results

### Measured and modelled photocurrent for human cones

We now compare the predictions of the model (Fig. 1B) with the human cone responses extracted using paired-flash ERG measurements [2]. This method was developed for studying rod responses [7,8], and has also been used to study the late phase of recovery of the human cone response to bright flashes [9,10].

**Figure 2:**
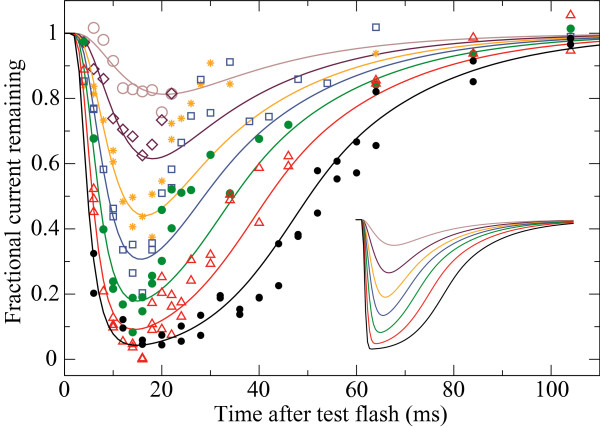
**Comparison of human photocurrent estimated from the ERG and predicted by the model, in response to flashes of different strength**. The symbols are taken from the figure numbered 9 in [2], and represent the normalized cone current obtained in paired-flash ERG measurements from the human eye. The measurements have been rescaled slightly (~ 5%) from [2], to compensate for what we presume to have been a small offset in the zero level of current. Thus the resting level (unity) has been retained unaltered, and a linear transformation has been applied so that a level of -0.05 in the above-mentioned figure of [2] has been converted to zero current here. The symbols employ the same colours as in the original figure, and represent the responses to brief flashes of the following intensities: 4 td s (gray open circles), 11 td s (purple diamonds), 22 td s (yellow asterisks), 37 td s (blue squares), 68 td s (green filled circles), 140 td s (red triangles), and 330 td s (black filled circles). The continuous curves of corresponding colour show the predictions of the model for the normalized current *J*(*t*)/*J*(0), using the parameter values listed in Table 1. The background intensity was set to 60 td, as measured for the blue rod-saturating background used in the ERG experiments. For comparison, the inset shows the curves predicted by the original model of [3] with the generic values for all parameters except the gain factor *k*_β_, which was reduced from 1.6·10^-4 ^(ms)^-1 ^td^-1 ^to 10^-4 ^(ms)^-1 ^td^-1^. In the present formulation, these curves correspond to setting τ_m _= 0, and represent *I*_os _in the notation of [3].

In Fig. 2, the symbols plot the experimental measurements presented in the figure numbered 9 in [2], for the fraction of cone circulating current remaining at a series of times after presentation of test flashes of different intensity, represented by the different colours. The curves of corresponding colours plot the predictions of the model illustrated in Fig. 1, for the flash intensities and background intensity (60 td) specified in [2] (see the legend of Fig. 2), using the parameters listed in Table 1.

We have made one adjustment to the data points, in the form of a small change to the zero level. The position of the zero level of cone current was estimated in [2] from the amplitudes of the responses to bright flashes, but in our preliminary fitting of the model we noticed a qualitative difference between the predictions and the measurements near the peak of the response, that we were able to eliminate by shifting the zero level of the cone circulating current by about 5%. Accordingly, we have altered the zero level for the symbols in Fig. 2 to correspond to a scale value of -0.05 in the corresponding figure of [2]; the dark current level has not been altered. This rescaling substantially improved the qualitative form of the fit, without significantly affecting the parameter values obtained from the fitting.

**Figure 3:**
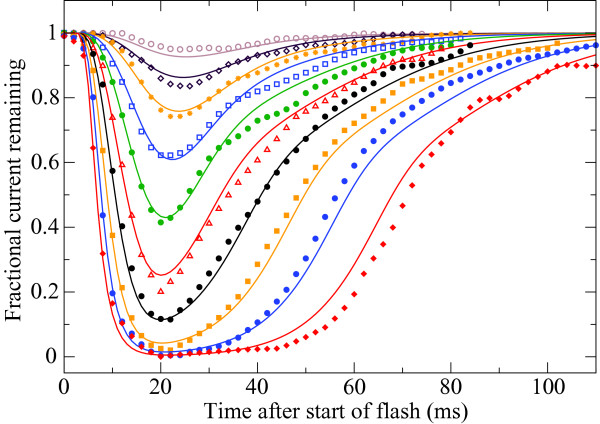
**Measured and modelled photocurrent of ground squirrel cones in response to flashes of different strength**. The symbols are sampled data points extracted from the curves plotted in the first figure of [13], and represent the cone current as a fraction of the circulating dark current (estimated as 67 pA from the fitting). The curves plot the predictions of the model for the normalized current *J*(*t*)/*J*(0), using the values of parameters listed in Table 1. The membrane capacitive time constant was set to τ_m _= 2.3 ms, while all other parameter values were obtained from the fitting. The parameter k_β _is specified in different units for this experiment, corresponding to the units in which the flash intensities were specified; these flash intensities increased in factors of 2 from 630 to 3.2·10^5 ^photoisomerizations per flash (using an effective collecting area of 0.64 μm^2 ^[13]). The flash duration was 11 ms, and here we have plotted the time origin with respect to the start of the flash (whereas it was plotted relative to the mid-point of the flash in [13]).

Between 20 and 35 ms after the flash, the symbols measured at the five lower flash intensities are reported to be affected by a 'labile spike' [2], an ERG component presumably generated by ON bipolar cells. Although efforts were made [2] to remove this artefact, the present model calculations suggest that this may not have been fully successful. Thus, the rapid recoveries of those responses differ from the predictions of the cone transduction model. We therefore tentatively consider those recovery phases to have been distorted, and we have consequently excluded the 20–35 ms region for these flash intensities from the fitting procedure. Subsequently, in Fig. 3, we examine this possibility further.

### Values of the parameters required in the fitting

A notable feature of the previous fitting of the model to macaque horizontal cell responses [3] is that three time constants were found to be very short. In the 'generic' set of parameters (Table 1 of [3]), the two time constants of active visual pigment lifetime and active transducin-PDE lifetime, τ_R _and τ_E_, were extracted as 3.4 and 8.7 ms (though it was not possible to determine which corresponded to which). The longer of these two time constants would determine the 'dominant time constant' of transduction, characterized by the shift in saturation time elicited by saturating flashes of increasing intensity [8]. The previous fitting indicated that another time constant, that of calcium extrusion (τ_Ca_), was also very small, at around 3 ms.

One time constant of the earlier fitting that was much longer (in darkness and in dim light) was the turnover time for cGMP, τ_β_, which exceeded 200 ms for backgrounds up to 10 td. The predicted light dependence of this time constant (which shortens considerably when transducin-PDE is activated; see [11]) provided an accurate explanation for the observed changes in integration time [4] when the background increased from 1 to 1000 td [3]. This change in integration time is not as large as the change in τ_β_, because the calcium control loop regulating the production of cGMP effectively produces a light-dependent high-pass filter that reduces the effect of changes in τ_β _[3].

When we fitted the model of transduction to the present data for human cone photoresponses, we chose to constrain as many as possible of the parameters to the 'generic' values determined in [3] for the macaque retina. Specifically, these standard values are as follows: τ_R _= 3.4 ms, τ_D _= 360 ms, *n*_X _= 1, τ_Ca _= 3 ms, *n*_cyc _= 4, and *a*_cyc _= 9·10^-2 ^(see Table 1). We set the outer segment membrane capacitive time constant to the mean value reported recently for primate cones, of τ_m _= 2.3 ms [12], but we found that values of τ_m _between 1 and 3 ms yielded fits of comparable quality. The three remaining parameters of the model (and the scaling factor for the zero level of cone current) were then optimized to provide the best fit of the model to experiment. As may be seen from Fig. 2, the resulting fit appears very satisfactory, and the required values of parameters (τ_E _= 9.6 ms, *k*_β _= 10^-4 ^(ms)^-1 ^td^-1^, and *t*_delay _= 1.3 ms; see Table 1) appear entirely reasonable.

The longer of the two inactivation time constants (of R* and E*) is the 'dominant time constant' [8] that determines the shift in saturation time with increasing intensity, and thereby in particular sets the relative positions of the curves for the two highest flash intensities in Fig. 2. The value obtained here, τ_E _= 9.6 ms, is only slightly larger than the 'generic' estimate τ_E _= 8.7 ms of [3], and well within the range reported there (τ_E _= 3.0–16.8 ms).

The coupling gain from light intensity to rate of hydrolysis of cGMP, *k*_β _= 10^-4 ^(ms)^-1 ^td^-1^, is only marginally smaller than the 'generic' estimate obtained in [3], 1.6·10^-4 ^ms^-1 ^td^-1^, and well within the range (of 0.5–4· 10^-4 ^ms^-1 ^td^-1^) reported in that study. Given the uncertainties of light intensity calibration in different studies, together with a possible difference in the troland conversion factor expected on the basis of different eye size between macaque and human, the similarity is impressive.

The overall delay time, *t*_delay _= 1.3 ms, reflects the sum of all delays not explicitly incorporated in the model, including the time for formation of active metarhodopsin, diffusional delays, and any electrical filtering delay in the instrumentation. Our value is somewhat shorter than the value of ~ 3 ms reported in [3], but this is not unexpected, given that that value also includes delays occurring in the cone inner segment, synapse, and horizontal cell.

The time constant of calcium equilibration, τ_Ca_, was fixed here at the generic value τ_Ca _= 3 ms of [3], but we found that for the present set of experiments similar fits could be obtained for any value of τ_Ca _smaller than about 12 ms. Larger values produced significantly increased RMS errors in the fits, and eventually biphasic responses.

Even without varying any parameter values, we found that the curves predicted by the original model (with its generic parameters) provided an adequate qualitative fit to the measurements, as illustrated by the inset in Fig. 2. These traces plot the predictions for *I*_chan _(corresponding to *I*_os _in [3]), which would equal the ERG-generating circulating current if the outer segment capacitive time constant were zero (τ_m _= 0). Note that in [3], the low-pass filtering attributable to the outer segment capacitance was not separately modelled, but was combined with the low-pass filtering caused by the cone inner segment, axon, and pedicle; the τ_m _in [3] is thus defined slightly differently from the τ_m _here. The only parameter that we allowed to vary for the inset of Fig. 2 was *k*_β_, the scaling factor linking incident light intensity to cGMP hydrolytic activity; this was set to the same value as in the main panel (see legend).

### Comparison with responses from ground squirrel cones

A potential criticism of the fitting illustrated in Fig. 2 is that it does not describe the rapid phase of recovery seen in some of the traces. Although the responses are well described over the rising phase and near the peak, and during the recovery following intense flashes, the description tends to be poorer during the recovery from flashes of intermediate intensity. As stated previously, we interpret this discrepancy to arise from a distortion of the ERG measurements caused by a residual component of the *b*-wave. To investigate that proposition, we now examine single-cell recordings obtained using the suction pipette technique, because these should be completely unaffected by post-receptoral activity. We have chosen to illustrate responses displaying the closest kinetics to the human ERG measurements that we have been able to find.

Fig. 3 illustrates a family of flash responses recorded by Kraft [13] from a ground squirrel cone using the suction pipette method. The experimental measurements (symbols) bear remarkable similarity to those for human cones extracted from the ERG. The time-to-peak for the dim flash response is around 20 ms after the mid-point of the flash (which had a duration of 11 ms in this experiment). And the qualitative form of the responses is also very similar, with a fast and monophasic time-course.

The same model as formulated in [3] and applied in Fig. 2 provides an excellent description of the responses, over the full range of flash intensities (curves). As indicated in the final column of Table 1, the values of parameters required for the fit are, for the most part, close to those required for human cones. (Note though that *k*_β _is expressed in different units, corresponding to the units of light intensity used in this study.) The most significant difference relates to the time constant of cGMP hydrolysis in darkness, τ_D_, which is 60 ms for the responses in Fig. 3, compared with 360 ms for the responses on human and macaque cones in Fig. 2 and in [3]. We suspect that this is a reflection of the fact that isolated cones exhibit a finite level of opsin dissociated from chromophore [14] and hence behave as light-adapted in comparison with cones in the intact retina/RPE.

It should be noted that, because the experimental data plotted in Fig. 3 were available at just a single background intensity (of zero), there are fewer constraints on the fitting of parameters than in the earlier study [3] where the values for the primate retina were determined over a range of background intensities. For the ground squirrel measurements presented here, it was possible to achieve similar fits with somewhat different values for the parameters; accordingly the values listed in the final column of Table 1 should be regarded as indicative rather than unique.

We interpret the results in Fig. 3 to indicate (*i*) that the apparent discrepancies in the fits in Fig. 2 are likely to have resulted from post-receptoral signal intrusion during the recovery phase following moderate flashes, (*ii*) that rapid monophasic responses may be a general property of mammalian cones, and (*iii*) that the model described here provides a good fit to ground squirrel cones as well as to human cones.

## Discussion

The cone photocurrent derived from the paired-flash ERG measurements in the human eye is reasonably well described by the phototransduction model presented here, using parameter values very similar to those obtained earlier from measurements in the macaque retina. The correspondence between these disparate approaches strengthens the claim that they both provide an adequate description of the cone photoresponse in the intact primate eye. The photoresponse to light flashes is characterized by fast dynamics (the response peaks in about 20 ms) and a simple, monophasic response shape.

In addition we have also shown that the model describes single-cell measurements of photocurrent in cones of the ground squirrel [13], with parameter values closely similar to those used for the macaque and for the human ERG (Table 1).

Certain limitations of the experimental recordings should be borne in mind. Firstly, the ERG recordings are obtained from the entire eye and hence represent a spatial average across the retina; therefore, if the response kinetics vary with eccentricity, a weighted average will have been recorded. Secondly, the ERG measurements appear to suffer from a residual component of *b*-wave response that has not been eliminated by the paired-flash method. In view of this shortcoming, we removed the region 20–35 ms after the flash from the fitting procedure. Thirdly, the single-cell recordings are obtained from a different mammalian species (ground squirrel) and therefore may have different properties. And finally, the single-cell recordings appear slightly light-adapted, probably as a result of the presence of a low level of unregenerated opsin [14].

The phototransduction model used here is consistent with the standard model developed to describe phototransduction in rods and cones (reviewed in [6]). However, the parameter values used for fitting both the present human ERG data and the earlier macaque measurements differ from typical rod values in several respects. Possibly the most important difference is that primate cones exhibit several very short time constants for recovery processes: the inactivation time constants of visual pigment and transducin-PDE are of the order of 3–10 ms, and the time constant for calcium equilibration is similarly short.

In order to obtain monophasic flash responses, the calcium equilibration time constant needs to be no larger than the inactivation time constants; a longer calcium time constant inevitably produces biphasic flash responses [3]. How could this turnover time for free calcium concentration be so much smaller in human cones (< 12 ms) than in amphibian cones (~ 100 ms, [15]), human rods (~ 100 ms [16]) or amphibian rods (400–500 ms, [17,18])? We suggest that the combination of a large surface-to-volume ratio (resulting from the cone sac structure) together with a low calcium buffering power in the outer segment enables the turnover time to be made extremely short.

The apparent Hill coefficient of CNG channel activation (here denoted by *n*_X_, but see below), and the Hill coefficient of GC activation (*n*_cyc_) had to be set at values close to those used for the macaque data (*n*_X _= 1 and *n*_cyc _= 4) in order to obtain good fits. Leaving both parameters free in the fit produced *n*_X _= 1.2 and *n*_cyc _= 4.4, with reasonable fits produced by the ranges *n*_X _= 1–1.5 and *n*_cyc _= 3–5. Outside these ranges the RMS error of the fits increased by more than 50%. The generic value of *n*_cyc _= 4 is larger than earlier reports of *n*_cyc_≅ 2 for rods (reviewed in [6]), but is consistent with a recent estimate [19].

The standard value of the Hill coefficient of the CNG channels is *n*_cG _= 3, which is clearly larger than the *n*_X _= 1–1.5 obtained here. The reason for this discrepancy is not clear, but one possibility is that it reflects the calcium modulation of the CNG channels [20], which effectively acts as a local negative feedback (denoted by the dashed lines in Fig. 1). The precise nature of this feedback is not known in human cones. Assuming that the feedback acts as a fast divisive gain control, a linear dependence of the divisor on [Ca^2+^] predicts *n*_X _= *n*_cG_/2, and a quadratic dependence on [Ca^2+^] predicts *n*_X _= *n*_cG_/3 [3]. In reality, the dependence on [Ca^2+^] will be more complicated than purely linear or quadratic, but the argument shows that a reduction of the CNG Hill coefficient *n*_cG _= 3 to an apparent Hill coefficient *n*_X _= 1–1.5 is within the range that might be produced by a local calcium feedback onto the channels.

The main characteristics of the flash response measured from the ERG and predicted by the model are fast dynamics and monophasic responses. These characteristics are different from those reported for isolated primate photoreceptors recorded using the suction pipette method [1,21]. Those responses showed considerably longer times-to-peak: typically > 50 ms, compared with about 20 ms for the present measurements and model. For background intensities lower than the 60 td used in the experiments simulated here, the time-to-peak of the dim-flash response is predicted by the model to increase to 31 ms at a background of 1 td, still considerably shorter than 50 ms. A second discrepancy is the biphasic shape of the flash response reported for isolated photoreceptors [1,21], whereas the present measurements and model simulations show essentially monophasic responses. The reasons for these discrepancies are not clear, but the latter would appear to be consistent with differences in the dynamics of the calcium feedback between the isolated cone preparation on the one hand, and the intact human eye and macaque retina-RPE-choroid preparation on the other hand.

## Conclusion

The close correspondence between the results obtained with the paired-flash ERG method from the human eye and the predictions of a model originally validated for measurements from the macaque retina supports the notion that both approaches provide accurate estimates of the cone phototransduction current. Furthermore, the close similarity of responses obtained from single cones in another mammalian preparation (ground squirrel) validates our assertion that the slight discrepancy in the ERG measurements arises from intrusion of a post-receptoral signal. The main characteristics of the human cone photocurrent are its rapid dynamics and its monophasic shape in response to brief flashes.

## Methods

### Electrophysiological measurements

The measurements illustrated in Fig. 2 are taken from the figure numbered 9 in [2]. In those experiments the size of the human cone circulating current was estimated using the paired-flash ERG technique, in which a saturating 'probe' flash was delivered at a range of intervals after a 'test' flash of required intensity. The pupil was dilated, and the rod response was suppressed by a steady blue background. For the cone system, the effective pupil area was taken as 20 mm^2^. See [2] for full details.

The measurements illustrated in Fig. 3 have been digitized from the first figure of [13]. In those experiments the circulating current of an isolated ground squirrel cone was measured using the suction pipette technique, and flashes at a range of intensities were delivered. Values were extracted graphically using public domain software (g3data). The measurements were then shifted by 5.5 ms to bring the time origin to the start of the 11 ms flash, and thereafter the values were interpolated at intervals of 2 ms.

### Model: Equations and calculations

#### Differential equations

The differential equations for the model are listed below. The variables used are: *t *is time; *I*(*t*) is the light intensity; R*(*t*) is quantity of R*; E*(*t*) is quantity of E*; *cG*(*t*) is concentration of cGMP; *Ca*(*t*) is free concentration Ca^2+^; *I*_chan_(*t*) is the ion current through the outer segment channels; *J*(*t*) is the externally recorded current; α(*t*) is the guanylate cyclase rate; and β(*t*) is the rate constant of hydrolysis of cGMP by PDE. Note that all variables are scaled according to the convention in [3]. The parameters used are defined in Table 1.

τ_R _dR*(*t*)/d*t *= *I*(*t*) - R*(*t*)     (1)

τ_E _dE*(*t*)/d*t *= R*(*t*) - E*(*t*)     (2)

d*cG*(*t*)/d*t *= α(*t*) - β(*t*) *cG*(*t*)     (3)

τ_Ca _d*Ca*(*t*)/d*t *= *I*_chan_(*t*) - *Ca*(*t*)     (4)

τ_m _d*J*(*t*)/d*t *= *I*_chan_(*t*) - *J*(*t*)     (5)

where

β(*t*) = 1/τ_D _+ *k*_β_E*(*t*)     (6)

α(t)=1/{1+[acyc Ca(t)]ncyc}     (7)
 MathType@MTEF@5@5@+=feaafiart1ev1aaatCvAUfKttLearuWrP9MDH5MBPbIqV92AaeXatLxBI9gBaebbnrfifHhDYfgasaacH8akY=wiFfYdH8Gipec8Eeeu0xXdbba9frFj0=OqFfea0dXdd9vqai=hGuQ8kuc9pgc9s8qqaq=dirpe0xb9q8qiLsFr0=vr0=vr0dc8meaabaqaciaacaGaaeqabaqabeGadaaakeaacqaHXoqydaqadaqaaiabdsha0bGaayjkaiaawMcaaiabg2da9iabigdaXiabc+caViabcUha7jabigdaXiabgUcaRiabcUfaBjabdggaHnaaBaaaleaacqqGJbWycqqG5bqEcqqGJbWyaeqaaOGaeeiiaaIaem4qamKaemyyae2aaeWaaeaacqWG0baDaiaawIcacaGLPaaacqGGDbqxdaahaaWcbeqaaiabd6gaUnaaBaaameaacqqGJbWycqqG5bqEcqqGJbWyaeqaaaaakiabc2ha9jaaxMaacaWLjaWaaeWaaeaacqaI3aWnaiaawIcacaGLPaaaaaa@50C7@

Ichan(t)=cG(t)nX     (8)
 MathType@MTEF@5@5@+=feaafiart1ev1aaatCvAUfKttLearuWrP9MDH5MBPbIqV92AaeXatLxBI9gBaebbnrfifHhDYfgasaacH8akY=wiFfYdH8Gipec8Eeeu0xXdbba9frFj0=OqFfea0dXdd9vqai=hGuQ8kuc9pgc9s8qqaq=dirpe0xb9q8qiLsFr0=vr0=vr0dc8meaabaqaciaacaGaaeqabaqabeGadaaakeaacqWGjbqsdaWgaaWcbaGaee4yamMaeeiAaGMaeeyyaeMaeeOBa4gabeaakmaabmaabaGaemiDaqhacaGLOaGaayzkaaGaeyypa0Jaem4yamMaem4raC0aaeWaaeaacqWG0baDaiaawIcacaGLPaaadaahaaWcbeqaaiabd6gaUnaaBaaameaacqqGybawaeqaaaaakiaaxMaacaWLjaWaaeWaaeaacqaI4aaoaiaawIcacaGLPaaaaaa@4378@

#### Steady background

The initial conditions, in the presence of a steady-state background of intensity *I*_B_, are given by

E*(0) = R*(0) = *I*_B _    (9)

J(0)=Ca(0)=Ichan(0)=cG(0)nX     (10)
 MathType@MTEF@5@5@+=feaafiart1ev1aaatCvAUfKttLearuWrP9MDH5MBPbIqV92AaeXatLxBI9gBaebbnrfifHhDYfgasaacH8akY=wiFfYdH8Gipec8Eeeu0xXdbba9frFj0=OqFfea0dXdd9vqai=hGuQ8kuc9pgc9s8qqaq=dirpe0xb9q8qiLsFr0=vr0=vr0dc8meaabaqaciaacaGaaeqabaqabeGadaaakeaacqWGkbGsdaqadaqaaiabicdaWaGaayjkaiaawMcaaiabg2da9iabdoeadjabdggaHnaabmaabaGaeGimaadacaGLOaGaayzkaaGaeyypa0JaemysaK0aaSbaaSqaaiabbogaJjabbIgaOjabbggaHjabb6gaUbqabaGcdaqadaqaaiabicdaWaGaayjkaiaawMcaaiabg2da9iabdogaJjabdEeahnaabmaabaGaeGimaadacaGLOaGaayzkaaWaaWbaaSqabeaacqWGUbGBdaWgaaadbaGaeeiwaGfabeaaaaGccaWLjaGaaCzcamaabmaabaGaeGymaeJaeGimaadacaGLOaGaayzkaaaaaa@4DC3@

where *I*_chan_(0) is obtained as the solution of the following equation (see Eq. 31 of the Supplementary Material in [3])

(1/τD+kβIB)Ichan(0)1/nX{1+[acycIchan(0)]ncyc}−1=0.     (11)
 MathType@MTEF@5@5@+=feaafiart1ev1aaatCvAUfKttLearuWrP9MDH5MBPbIqV92AaeXatLxBI9gBaebbnrfifHhDYfgasaacH8akY=wiFfYdH8Gipec8Eeeu0xXdbba9frFj0=OqFfea0dXdd9vqai=hGuQ8kuc9pgc9s8qqaq=dirpe0xb9q8qiLsFr0=vr0=vr0dc8meaabaqaciaacaGaaeqabaqabeGadaaakeaadaqadaqaaiabigdaXiabc+caViabes8a0naaBaaaleaacqqGebaraeqaaOGaey4kaSIaem4AaS2aaSbaaSqaaiabek7aIbqabaGccqWGjbqsdaWgaaWcbaGaeeOqaieabeaaaOGaayjkaiaawMcaaiabdMeajnaaBaaaleaacqqGJbWycqqGObaAcqqGHbqycqqGUbGBaeqaaOWaaeWaaeaacqaIWaamaiaawIcacaGLPaaadaahaaWcbeqaaiabigdaXiabc+caViabd6gaUnaaBaaameaacqqGybawaeqaaaaakiabcUha7jabigdaXiabgUcaRiabcUfaBjabdggaHnaaBaaaleaacqqGJbWycqqG5bqEcqqGJbWyaeqaaOGaemysaK0aaSbaaSqaaiabbogaJjabbIgaOjabbggaHjabb6gaUbqabaGcdaqadaqaaiabicdaWaGaayjkaiaawMcaaiabc2faDnaaCaaaleqabaGaemOBa42aaSbaaWqaaiabbogaJjabbMha5jabbogaJbqabaaaaOGaeiyFa0NaeyOeI0IaeGymaeJaeyypa0JaeGimaaJaeiOla4IaaCzcaiaaxMaadaqadaqaaiabigdaXiabigdaXaGaayjkaiaawMcaaaaa@6CB3@

#### Normalization

For plotting the responses in Figs 2 and 3, the current has been normalized to its steady level in the presence of the background; i.e. as *J*(*t*)/*J*(0). In Fig. 2 the background was dim, while in Fig. 3 the background was nominally zero, though the isolated cone behaved as if it was slightly light-adapted.

#### Flash

For fitting the ERG responses, which employed brief xenon flashes, the flash was treated as an impulse of 1 ms duration at *t *= 0, with an intensity of *Q*/1 ms (in td, with *Q *in td s). The resulting response was shifted by the delay time, *t*_delay_, by linear interpolation. For the Matlab routine the xenon flash was treated as an impulse of size *Q *(in td s) delivered at the delay time, *t*_delay_. In this case, the solution of eqn (1) is

R*(*t*) = *Q*/τ_R _exp(-(*t*-*t*_delay_)/τ_R_) + *I*_B _    for *t *≥ *t*_delay_.     (12)

#### Numerical integration

The differential equations were integrated numerically using two approaches. Firstly, the fitting was performed using a very fast algorithm, consisting of a series of autoregressive moving-average (ARMA) filters in accordance with the scheme in Fig. 1B, which contains only first-order low-pass filters and static nonlinearities. For a first-order low-pass filter with time constant *τ*, the output *y*(*n*) to an input *x*(*n*), with a time step Δ*t*, is given by the following ARMA filter [22]

*y*(*n*) = *f*_1 _*y*(*n *- 1) + *f*_2 _*x*(*n *- 1) + *f*_3 _*x*(*n*)     (13)

with

*f*_1 _= exp(-1/τ')

*f*_2 _= τ' - (1 + τ') exp(-1/τ')     (14)

*f*_3 _= 1 - τ' + τ' exp(-1/τ')

τ' = τ/Δ*t*.

In these ARMA calculations we used a time step of Δ*t *= 100 μs. See [3] for further details, including example Fortran90 code. In the second approach, the numerical integration routine 'ode45' in Matlab (The MathWorks) was used; a sample program is available from the authors. The results using the two approaches were indistinguishable, but the ARMA method was about 25-fold faster.

## Abbreviations

CNG: Cyclic nucleotide-gated

ERG: Electroretinogram

GC: Guanylyl cyclase

PDE: Phosphodiesterase

RMS: root-mean-square

RPE: Retinal pigment epithelium

## Authors' contributions

TDL proposed relating the ERG measurements to the phototransduction model, JHvH performed the calculations, and JHvH and TDL jointly interpreted the results and wrote the article.

## Figures and Tables

**Table 1:** Parameters of the model. Values used in the fits illustrated in Figs 2 and 3 are compared with the generic values for macaque reported in [3].

Parameter	Description	Generic macaque cone [3]	Human cone (Fig. 2)	Ground squirrel (Fig. 3)	Units
τ_R_	Time constant of R* inactivation	3.4	3.4	3.0	ms
τ_E_	Time constant of E* inactivation	8.7	9.6	13	ms
τ_D_	Time constant of cGMP hydrolysis in darkness	360	360	60	ms
*k*_β_	Coupling gain from light intensity to rate of hydrolysis of cGMP	1.6·10^-4^	10^-4^		(ms)^-1 ^td^-1 ^
				2.1·10^-7^	(ms)^-1 ^(isom/s)^-1^
*n*_X_	Apparent Hill coefficient of CNG channel activation	1	1	1.7	-
τ_Ca_	Time constant of Ca^2+ ^extrusion	3	3	4.8	ms
*n*_cyc_	Hill coefficient of GC activation	4	4	3.2	-
*a*_cyc_	Scaling constant of GC activation	9·10^-2^	9·10^-2^	3.7·10^-2^	-
τ_m_	Outer segment capacitive time constant	< 4	2.3	2.3	ms
*t*_delay_	Delay time	< 3	1.3	2.2	ms
